# Membrane and Extracellular Matrix Glycopolymers of *Colwellia psychrerythraea* 34H: Structural Changes at Different Growth Temperatures

**DOI:** 10.3389/fmicb.2022.820714

**Published:** 2022-02-25

**Authors:** Angela Casillo, Caterina D’Angelo, Ermenegilda Parrilli, Maria Luisa Tutino, Maria Michela Corsaro

**Affiliations:** Department of Chemical Sciences, University of Naples “Federico II,” Complesso Universitario Monte S. Angelo, Naples, Italy

**Keywords:** biofilm, cold-adapted bacterium, extracellular polysaccharides, temperature changes, structural characterization, CLSM

## Abstract

*Colwellia psychrerythraea* 34H is a marine Gram-negative psychrophile; it was isolated from Arctic marine sediments, but it is considered cosmopolitan in cold environments. This microorganism is considered a model to study adaptive strategies to sub-zero temperatures, and its lifestyle has been the object of numerous studies. In the last few years, we focused our studies on the glycoconjugates produced by *C. psychrerythraea* 34H at 4°C, resulting in the isolation and characterization of very interesting molecules. It produces an unusual lipooligosaccharide molecule and both capsular and medium released polysaccharides. In this study, we described the response of these glycoconjugates in terms of production and chemical structure produced by *C. psychrerythraea* 34H grown in planktonic conditions at −2, 4, and 8°C. The glycopolymers have been detected by chemical methods and spectroscopic analyses. Moreover, the glycopolymer content of the biofilm matrix of *C. psychrerythraea* 34H has been evaluated, through confocal microscopy and glycosyl analysis. The results highlighted that *C. psychrerythraea* 34H adjusts both the production and the typology of its glyconjugates in response to temperature fluctuations.

## Introduction

Arctic and Antarctic sea-ice, glaciers, and deep ocean waters are some of the examples of environments where the temperature is below 0°C. These habitats create the perfect niches for the colonization of psychrophiles, microorganism able to grow well at temperatures around the freezing point of water. These bacteria evolved several strategies to counteract the lowering temperatures (Siddiqui et al., 2013; Collins and Margesin, 2019). The main studies concerning the adaptation strategies are focused on the overexpression or the production of cold shock proteins, and ice-binding proteins (IBPs). Furthermore, the modulation of enzymes involved in synthesis of the membrane components, such as acyl chains of lipids, LPS, peptidoglycan, and outer membrane protein biosynthesis, is reported. Recently, the role of the surface glycoconjugates molecules in the cold adaptation mechanism has been investigated (Nichols et al., 2005; Carillo et al., 2015; Casillo et al., 2019a).

The outer membrane (OM) of Gram-negative bacteria is an asymmetric bilayer with the inner monolayer composed of phospholipids and the outer leaflet mainly composed of lipopolysaccharides (LPS). The main role of the OM is to protect the cell by forming a protective barrier (Nikaido and Vaara, 1985; Nikaido, 1994), which makes the bacteria resistant to a variety of host defense factors, antibiotics, and stress conditions (Daugelavičius et al., 2000; Guyard-Nicodème et al., 2008).

The LPSs are embedded in the OM through the lipid A domain, a glycolipid portion consisting of two phosphorylated glucosamines (GlcN) that carry long-chain fatty acids. In addition to this, the LPS contains two saccharide portions: the core oligosaccharide linked to the lipid A, and the O-polysaccharide region (O-chain) exposed to the outside. The last portion is responsible for the different phenotypes of Gram-negative bacteria. The smooth phenotype occurs when the O-chain is linked to the core, while the rough phenotype occurs when bacteria are unable to add the O-chain repeating unit to their outer core region. The alteration of cell membrane composition to preserve the membrane fluidity at low temperatures has been observed in terms of phospholipid composition (Russell, 1990; Ernst et al., 2016). Since the lipid A moiety represents a major part of the outer leaflet, its chemical structure is also crucial in the maintenance of the membrane integrity.

Finally, many of the LPSs characterized from cold-adapted bacteria lack the O-chain when grown at a temperature above 20°C (Casillo et al., 2019a). The investigation on the response of the cell membrane to lower temperature is well studied in mesophilic bacteria (Cronan and Gelmann, 1975; Janoff et al., 1979; Kropinski et al., 1987), while too few investigations are conducted on psychrophiles (Ray et al., 1994; Kumar et al., 2002; Corsaro et al., 2004). Additionally, some bacteria are reported to produce extracellular polysaccharides, strictly associated with the cell surface as a capsule (capsular polysaccharide) or totally released in the surrounding medium (medium released polysaccharide, MRP) (Casillo et al., 2018). Extracellular polysaccharides facilitate the adhesion on the biotic and abiotic surfaces, cellular aggregation, and nutrient trapping (Quintero and Weiner, 1995; Rinker and Kelly, 1996; Langille and Weiner, 1998)WeinerKelly, and confer cryoprotection to the bacterial cell (Kim and Yim, 2007; Marx et al., 2009; Carillo et al., 2015; Casillo et al., 2017a). Extracellular polysaccharides are also essential structural components of the biofilm matrix. Biofilm has been defined as an aggregate of microorganisms in which the cells are surrounded by a self-produced matrix of extracellular polymeric substances (EPS) (Flemming and Wingender, 2010). Although the precise chemical and physical composition of the EPS varies according to the species and the growth conditions, the main biofilm matrix building blocks are bacterial proteins, extracellular DNA (eDNA) (Whitchurch et al., 2002), lipids, and polysaccharides (Flemming and Wingender, 2010).

In the natural environment, the biofilm formation has several advantages, i.e., the matrix captures resources such as the nutrients that are present in the environment (Flemming and Wingender, 2010) and affords protection from a wide range of environmental challenges (Flemming et al., 2016), such as UV exposure, metal toxicity, dehydration, and salinity and phagocytosis. Therefore, the ability to form biofilm is a selective advantage for bacteria. Correspondingly, bacteria living in extreme environments, like the Polar region, can be found as biofilms, and this ability is believed to aid their adaptation and survival in the environment (Liao et al., 2016; Ricciardelli et al., 2018).

To deeply investigate the implication of carbohydrate-based molecules in cold adaptation mechanism, we have been interested in the structural elucidation of glycoconjugates isolated from the psychrophile *Colwellia psychrerythraea* 34H.

*C. psychrerythraea* 34H (henceforth *Colwellia* 34H) is a psychrophilic bacterium, originally isolated from subzero Arctic sediments (Methé et al., 2005), considered a cosmopolitan species to both poles and sea-ice (Boetius et al., 2015). *Colwellia* 34H is used as model species for investigating the metabolism of cold-adapted microbes. This bacterium can grow in a heterotrophic medium over a temperature range of −4 to 10°C, with an optimum of 8°C (Marx et al., 2009). Recently, the genome of *Colwellia* 34H was used as type strain to construct the whole genome phylogeny. The obtained phylogenomic trees showed that the genus *Colwellia* was paraphyletic, and that four members of the genus *Colwellia*, including *Colwellia* 34H, formed a clade (Liu et al., 2020).

In the last years, we focused our research on the elucidation of the surface carbohydrate molecules produced by the model psychrophile *Colwellia* 34H. The large-scale cultivation of *Colwellia* 34H at 4°C allowed to isolate and characterize very intriguing and original glycoconjugates moiety. The inner portion of the LPS, the lipid A, displays the presence of phosphoglycerol moiety that can be additionally acylated, resulting in a lipid A carrying up to seven fatty acids (Casillo et al., 2017b). The core oligosaccharide structure confers a high negative charge density to the entire molecule since it contains many acidic residues. In addition, it showed a phosphoglycerol, and the core structure ends with the rare sugar colitose (Carillo et al., 2013). *Colwellia* 34H produces both CPS and MRP (Carillo et al., 2015: Casillo et al., 2017a) decorated with amino acids, the structures of which resemble those of the antifreeze glycoproteins (AFGP). These polysaccharides, through their peculiar primary structures and three-dimensional conformation, inhibit the ice crystal growth, exhibiting their anti-freezing effect. Since *Colwellia* 34H genome lacks genes encoding for AFGP, this alternative mechanism to contrast the freezing of the cells makes these polymers even more fascinating. Finally, the large-scale cultivation of *Colwellia* 34H at 8°C allowed to isolate a CPS (Casillo et al., 2017c) containing all amino sugars.

The complex mechanism adopted by *Colwellia* 34H to face the low temperatures makes this bacterium the perfect candidate to get to the bottom of the understanding of cold adaptation.

In this paper, we analyzed the effect of the temperature changes on the production and the structures of the surface carbohydrate molecules, and on the biofilm produced by *Colwellia* 34H.

## Experimental Section

### Bacteria Growth in Planktonic and Sessile Conditions

*Colwellia* 34H was grown aerobically at −2, 4, and 8°C in Marine Broth medium (DIFCO 2216). Cells were harvested at different times of growth (48, 72, and 96 h) by centrifugation for 20 min at 5,000 rpm and 4°C. The biofilm formation was assessed in the static condition in the Marine Broth medium at 8 and 4°C for 48 h while −2°C for 96 h. The kinetic of the biofilm formation was performed at 4°C at different times (24, 48, 72, 96, and 120 h).

### Transmission Electron Microscopy Images

The samples were prepared for TEM observations as detailed in Escalera et al. (2014). Briefly, specimens were fixed with 2.5% glutaraldehyde, post-fixed with 1% osmium tetroxide, dehydrated in a graded ethanol series further substituted by propylene oxide, and embedded in Epon 812 (TAAB, TAAB Laboratories Equipment Ltd., Berkshire, United Kingdom). Ultrathin sections (60 nm thick) were collected on nickel grids and stained with uranyl acetate and lead citrate, and transmission electron microscopy (TEM) images were acquired with a Zeiss LEO 912AB TEM (Zeiss, Oberkochen, Germany) operating at an accelerating voltage of 80 kV.

### Lipopolysaccharides Isolation Purification

Dried cells were extracted three times with a mixture of aqueous phenol 90%/chloroform/light petroleum ether (2:5:8) (PCP) as described by Galanos et al. (1969). After removal of the organic solvents under vacuum, LPS was precipitated from phenol with drops of water, then washed with cold acetone, and then lyophilized (LPS yields: 1.1, 3, and 0.6% at −2, 4, and 8°C, respectively).

#### Lipid A Isolation

The PCP extracts were hydrolyzed with 1% CH_3_COOH (10 mg/ml, 100°C for 3 h). The obtained suspensions were then centrifuged (7,000 rpm, 4°C, 30 min) (Casillo et al., 2017b). The precipitates containing the lipid A were washed several times with chloroform/methanol (1:2) mixture to obtain purified samples and then analyzed by GC-MS.

#### Core Oligosaccharide Isolation

An aliquot of the PCP extracts was incubated with hydrazine at 37°C (20–30 mg/ml, 1.5 h). The precipitation of the *O*-deacylated LOSs was achieved by adding cold acetone and recovered after centrifugation (4°C, 7,000 rpm, 30 min). The LOS-OH samples were lyophilized, and then dissolved in 4 M KOH (120°C for 16 h). The mixtures were neutralized and extracted three times with CHCl_3_, and finally desalted on a Sephadex G-10 column (Amersham Biosciences, 2.5 × 43 cm, 35 ml/h, fraction volume 2.5 ml, eluent NH_4_HCO_3_ 10 mM). The eluted oligosaccharide mixtures were then lyophilized.

### Capsular Polysaccharides Isolation and Purification

The cell residues after PCP extraction were extracted by phenol/water method according to Westphal and Jann (1965) procedure. The cells were suspended in aqueous phenol/water 90%/(1:1 v/v) at 68°C as already reported. The collected water phases were dialyzed against water (cutoff 3,500 Da) and freeze-dried. The extracts were subjected to enzymatic digestion with DNase, RNase, and protease K (Sigma-Aldrich) to remove nucleic acids. The water extracts were hydrolyzed with 1% aqueous CH_3_COOH (10 mg/ml, 100°C, 3 h), and centrifuged (7,000 rpm, 4°C, 30 min). The supernatant portion was fractionated on a Sephacryl S-400HR (Sigma, 0.5 × 110 cm, fraction volume 2.5 ml) eluted with 0.05 M ammonium hydrogen carbonate, to isolate the polysaccharidic material (CPS yields:1.4, 1.6, and 1.2% at −2, 4, and 8°C, respectively).

### Medium Released Polysaccharide Isolation and Purification

The cell-free supernatants were dialyzed against water (cutoff 3,500 Da) and lyophilized, and subsequently hydrolyzed with 5% aqueous CH_3_COOH (100°C, 3 h). The resulting suspensions were centrifuged (7,000 rpm, 4°C, 30 min), and the supernatant portions were then fractionated on a Sephacryl S-400 HR column (Sigma, 1.5 × 95 cm, flow 16.8 ml/h, fraction volume 2.5 ml), eluted with 0.05 M ammonium hydrogen carbonate. The obtained polysaccharides were further analyzed by chemical analyses.

### Chemical Analyses

Monosaccharides were analyzed as acetylated methyl glycoside (AMG) derivatives as reported (Adinolfi et al., 1996). Briefly, 0.5–1 mg of sample was subjected to a methanolysis reaction (1.25 M HCl/MeOH, 1 ml, 80°C, 16 h). The obtained *O*-methyl-glycosides were extracted three times with hexane and the methanol layers were acetylated with Ac_2_O (50 μl) and Py (50 μl) at 100°C for 30 min and analyzed by using an Agilent Technologies gas chromatograph 7,820A equipped with a mass selective detector 5977B and an HP-5 capillary column (Agilent, 30 m × 0.25 mm i.d.; flow rate, 1 ml/min, He as carrier gas). The following temperature program was used for the analysis of the AMG: 140°C for 3 min, 150°C → 240°C at 3°C/min. The hexane layer containing fatty acid methyl esters (FAME) were analyzed by GC-MS to obtain fatty acid composition, by using the following temperature program: 140°C for 3 min, 140°C → 280°C at 10°C/min, and 280°C for 20 min.

### Deoxycholate-PAGE

PAGE was performed using the system of Laemmli (Laemmli and Favre, 1970) with sodium deoxycholate (DOC) as the detergent, as already described (Casillo et al., 2019b). The gels were fixed in an aqueous solution of 40% ethanol and 5% acetic acid and visualized after Alcian Blue and silver nitrate staining (Tsai and Frasch, 1982).

### MALDI TOF/TOF

MALDI-TOF mass spectra were acquired on an ABSCIEX TOF/TOF™5800 (AB SCIEX, Darmstadt, Germany). The core oligosaccharide samples were desalted on a Dowex 50WX8 (H + form) and dissolved in 2-propanol/water (v/v 1:1). The 2, 5-dihydroxybenzoic acid (DHB) dissolved in 20% CH_3_CN in water (25 mg/ml) was used as matrix. The spectra were calibrated with a hyaluronan oligosaccharide mixture and processed under computer control by using Data Explorer software v.0.2.0 (Microsoft, Albuquerque, NM, United States).

### Biofilm

#### Biofilm Formation and Assays

The quantification of the *in vitro* biofilm production was based on the method described by Christensen with slight modifications (Christensen et al., 1999). Briefly, the wells of a sterile 24-well flat-bottomed polystyrene plate were filled with 1 ml of a medium with a suitable dilution of the Artic bacterial culture in the exponential growth phase (about 0.2 OD 600 nm). For the analysis of the temperature effect on *Colwellia* 34H biofilm formation, the plates were incubated at −2°C for 96 h, and at 4 and 8°C for 48 h, while for the kinetics of the biofilm formation, the plates were incubated at 4°C for different times (24, 48, 72, 96, and 120 h). After rinsing with PBS, the adherent cells were stained with 0.1% (w/v) crystal violet, rinsed twice with double-distilled water, and thoroughly dried. Subsequently, the dye bound to the adherent cells was solubilized with 20% (v/v) acetone and 80% (v/v) ethanol. After 10 min of incubation at room temperature, the OD 590 nm was measured to quantify the total biomass of biofilm formed in each well. Each data point was composed of six independent samples.

#### Biofilm Recovery

The biofilm formation at the air–liquid interface was assessed in the Marine Broth medium at 4°C. Briefly, the wells of a sterile 24-well flat-bottomed polystyrene plate were filled with 1 ml of a medium with a suitable dilution of a culture of *Colwellia* 34H in the exponential growth phase (about 0.2 OD 600 nm). The plates were incubated at 4°C for 120 h to allow the formation of compact and resistant pellicles at the air–liquid interface. After incubation, the pellicles were recovered using a pipette and stored at −20°C. The samples were freeze-dried for further analysis.

#### DNase I and Proteinase K Effects on Biofilm Formation

To understand if DNase I and proteinase K can affect the *Colwellia* 34H biofilm formation process, a static biofilm assay was performed in the presence of DNase I or proteinase K. The biofilm formation was assessed in Marine Broth medium at 4°C for 96 h. In detail, 200 μl of the medium with a suitable dilution of *Colwellia* 34H culture in the exponential growth phase (about 0.2 OD 600 nm) were added into each well of a sterile 96-well flat-bottomed polystyrene plate in the absence and presence of DNase I or proteinase K (100 μg/ml). After incubation, biofilm quantification was performed by means of the crystal violet method, as previously described (Christensen et al., 1999).

### Confocal Laser Scanning Microscopy

For the confocal microscopy analysis, the biofilm formation was performed on Nunc™ Lab-Tek^®^ 8-well Chamber Slides (n° 177445; Thermo Scientific, Ottawa, ON, Canada) in Marine Broth medium at 8 and 4°C for 72 h while at −2°C for 96 h. All the microscopic observations and image acquisitions were performed with a confocal laser scanning microscope (CLSM) (LSM700-Zeiss, Germany) equipped with an Ar laser (488 nm), and a He-Ne laser (555 nm). The biofilm observed by CLSM was formed at the bottom of the well.

#### Bacterial Viability and Biofilm Thickness Determination

The biofilm cell viability was determined with the FilmTracer™ LIVE/DEAD^®^ Biofilm Viability Kit (Molecular Probes, Invitrogen) following the manufacturer’s instructions. Briefly, 300 μl of the medium with a suitable dilution of a *Colwellia* 34H culture in the exponential growth phase (about 0.2 OD 600 nm) were added into each well of a sterile Chamber Slide. After the incubation, the plates were rinsed with filter-sterilized PBS. Then, each well of the chamber slide was filled with 300 μl of a working solution of fluorescent stains, containing the SYTO 9 green-fluorescent nucleic acid stain (10 μM) and propidium iodide, the red-fluorescent nucleic acid stain (60 μM), and incubated for 20–30 min at room temperature, protected from light. All the excess staining was removed by rinsing gently with filter-sterilized PBS. The Z-stacks images were obtained using a 20X NA 0.8 objective and acquired using these parameters: scaling (per pixel) of 63 (X) × 0.63 (Y) × 1 μm (Z); image size (pixels) of 512 × 512; bit depth of 8 bit; pixel dwell of 1.58 μm. The excitation/emission maxima for these dyes are approximately 480/500 nm for the SYTO^®^ 9 stain and 490/635 nm for propidium iodide. Z-stacks (XYZ isosurface) were obtained by driving the microscope to a point just out of focus on both the top and the bottom of the biofilms with a step size of 1 μm. The images were analyzed with ZEN black Imaging Software 3.0 and recorded as a series of. tif files with a file depth of 16 bits. The COMSTAT software package (Heydorn et al., 2000) was used to determine the biomasses (μm^3^/μm^2^), mean thicknesses (μm), and roughness coefficients (Ra*). For each condition, two independent biofilm samples were used.

#### Qualitative Imaging of the Matrix Components

To identify the polysaccharide and protein contents of the biofilm matrix, the biofilms were labeled with wheat germ agglutinin (WGA), from Triticum vulgaris, FITC conjugated (L4895; Sigma-Aldrich), which binds di-*N*-acetylglucosamine and *N*−acetylneuraminic acid (415/518 nm), and with FilmTracer™ SYPRO^®^ Ruby Biofilm Matrix stain (Molecular Probes, Invitrogen), which labels most classes of proteins including glycoproteins, phosphoproteins, lipoproteins, calcium-binding proteins, fibrillar proteins, and other proteins that are difficult to stain (450/610 nm). Briefly, 300 μl of the medium with a suitable dilution of the Arctic culture in the exponential growth phase (about 0.2 OD 600 nm) were added into each well of a sterile Chamber Slide. After the incubation, the plates were rinsed with filter-sterilized PBS. Next, each well of the chamber slide was filled with 300 μl of a working solution of fluorescent stains (WGA or SYPRO Ruby), following the manufacturer’s instructions, and incubated for 20–30 min at room temperature, protected from light. All the excess staining was removed by rinsing gently with filter-sterilized PBS. The 2D images (“snap”) were obtained using a 20 × NA 0.8 objective and acquired using these parameters: scaling (per pixel) of 63 μm (X) × 0.63 μm (Y); image size (pixels) of 512 × 512; bit depth of 8 bit; pixel dwell of 1.58 μm. Then, the images were analyzed with ZEN black Imaging Software 3.0 and recorded as a series of. tif files with a file depth of 16 bits. For each condition, two independent biofilm samples were used.

## Results

### Bacteria Growth Conditions

*C. psychrerythraea* strain 34H was grown aerobically at −2, 4, and 8°C in the Marine Broth medium, and the cells were separated from spent medium by a gentle centrifugation and freeze-dried. The supernatants were dialyzed against water and lyophilized. The obtained biomasses for all the conditions are reported in Supplementary Table 1.

As previously performed in case of *Colwellia* 34H grown at 4°C, a preliminary analysis on the Arctic bacterium grown at −2 and 8°C was performed by transmission electron microscopy, and the presence of a capsular structure around the cells was visible either at −2 or at 8°C (Supplementary Figure 1).

### Lipopolysaccharides Isolation, Purification, and Characterization

**FIGURE 1:**
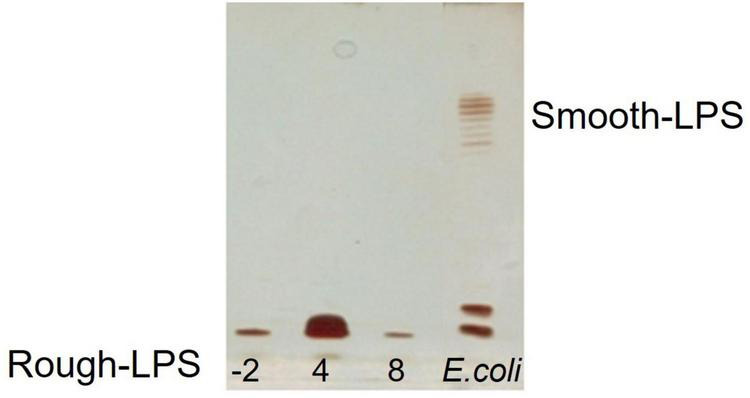
DOC-PAGE analysis of the PCP extracts from *Colwellia* 34H cells grown at −2, 4, and 8°C.

The dried cells from *Colwellia* 34H grown at the temperatures of −2, 4, and 8°C were extracted by the PCP method to recover the crude LPSs. The DOC-PAGE analysis, visualized after silver nitrate (Figure 1), confirmed that *Colwellia* 34H produces a rough-LPS (LOS) in all the conditions, as shown by the absence of the typical ladder-like pattern in the upper part of the gel attributable to the O-chain moiety. Moreover, differences in the yield of the produced LPSs at the three different temperatures were observed (yields: −2°C 1.1%; 4°C 3%; 8°C 0.6%).

Chemical analyses performed on the pure LOS for each growth revealed the presence of colitose, glucuronic acid, mannose, and glucosamine. The presence of Kdo in the three samples was confirmed after the HF treatment.

#### Lipid A

The intact LOS extracts were subjected to mild acid hydrolysis with acetic acid to separate the lipid A portion from the saccharidic one. The mixtures were centrifuged, and the precipitates were freeze-dried, and then washed several times with a chloroform/methanol mixture to obtain the purified samples. The analysis of fatty acid composition was performed for each sample and the results are reported in Table 1. The data suggested that *Colwellia* 34H exhibited slight changes in fatty acid composition in response to the growth temperature. The growth at −2°C resulted in an increased percentage of mono-unsaturated fatty acids (C16:1), confirming the role of the lipid A in the modulation of membrane fluidity. In contrast with the results obtained for *P. syringae*, in *Colwellia* 34H, the hydroxylated acyl chains do not seem to participate in the adaptation mechanism since the highest amount of the last was found for *Colwellia* 34H grown at 8°C (27.25%). Finally, we observed that saturated fatty acids did not change significantly. Although the obtained results in this study are counterintuitive when compared with those reported for mesophilic bacteria, they agree with the findings reported for other cold-adapted bacteria. It is also evident that if we limit our analysis by considering only the temperature of −2 and 4°C, the variations in unsaturation, hydroxylation, and acyl chain length are all in agreement with the available data. A similar response was also observed for *P. haloplanktis* TAC 125. This uncommon trend could find a right explanation in the capacity of *Colwellia* 34H to grow at 8°C, which probably represents a limit in growth temperature in the lab, whereas −2 and 4°C are close to those of the natural environments.

**TABLE 1:** Temperature dependence in fatty acid composition reported for *Colwellia* 34H.

*Growth temperature (°C)*	*−2*	*4*	*8*
***Fatty acid***	**Fatty acid composition (%)**		
*C10:0*	8.33	8.84	9.72
*C12:0*	13.40	9.01	13.09
*C14:0*	2.87	4.10	3.24
*C15:0*	0.61	0.75	0.05
*C16:0*	32.85	41.98	30.36
***Sum saturated***	**58.08**	**64.68**	**56.46**
*C14.1*	4.61	5.26	5.20
*C15:1*	0.53	0.70	1.07
*C16:1*	15.37	10.05	10.02
***Sum monounsaturated***	**20.51**	**16.01**	**16.29**
*C12:3OH*	19.38	16.12	22.73
*C14:3OH*	0.12	0.67	0.69
*C14:1 3OH*	1.92	2.52	3.83
***Sum hydroxylated***	**21.41**	**19.31**	**27.25**

#### Core Oligosaccharide Structure

**FIGURE 2:**
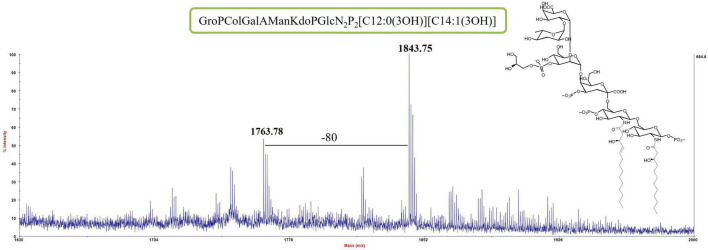
MS spectrum and chemical structure of the partially deacylated LOS (LOS-OH) from *Colwellia* 34H grown at 8°C.

An aliquot of each LOS sample was deacylated with anhydrous hydrazine. The LOS-OH product obtained from both samples was analyzed by MALDI MS. The partially deacylated LOS (LOS-OH) obtained for both LOS from cells grown at −2°C and 8°C revealed the identical composition with the LOS from *Colwellia* 34H at 4°C (Figure 2 and Supplementary Figure 2).

The LOS-OH were completely deacylated by strong alkaline hydrolysis and purified by gel filtration chromatography. The resulting core oligosaccharides (OSs) were analyzed by NMR experiments.

**SCHEME 1:**
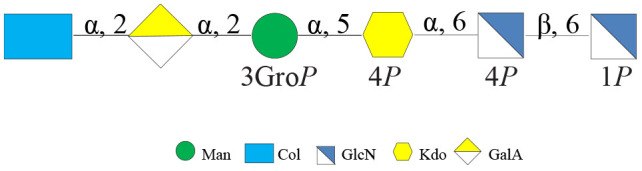
Core oligosaccharide structure from *Colwellia* 34H.

Both MS results of the LOS-OH and ^1^H NMR spectra of the OS (Supplementary Figure 3) revealed that *Colwellia* 34H produces the same core oligosaccharide structure at all temperatures (Scheme 1).

### Capsular Polysaccharides

The phenol/water extracts were analyzed by GC-MS. The samples derivatized as AMG suggested the presence of glucuronic (GlcA) and galacturonic (GalA) acids, galactosamine (GalN), and glucosamine (GlcN), together with traces of mannose (Man) in all the tested conditions. Finally, at 8°C, traces of glucosaminuronic acid (GlcNA) were found. The analysis of the monosaccharide composition confirmed the production of the CPS_*A*_ isolated from the large-scale cultivation at 4°C. Furthermore, the analysis also revealed the production of the CPS_*B*_ only at 8°C. Similarly to the fatty acids, the effect of the growth temperature on the percentage composition of monosaccharides was evaluated (Table 2).

**TABLE 2:** Temperature dependence in monosaccharide composition reported for *Colwellia* 34H.

Growth temperature (°C)	−2	4	8

	**Monosaccharide composition (%)**		
GlcA	14,18	15,23	17,68
GalA	33,24	27,06	24,25
GalN	16,13	15,59	16,25
GlcN	36,45	42,12	41,82
GlcNA	−	−	Traces

*GlcA, CPS_A_; GalA, CPS_A_ and LPS; GalN, CPS_A_ and CPS_B_; GlcN, CPS_A_ and LPS.*

**FIGURE 3:**
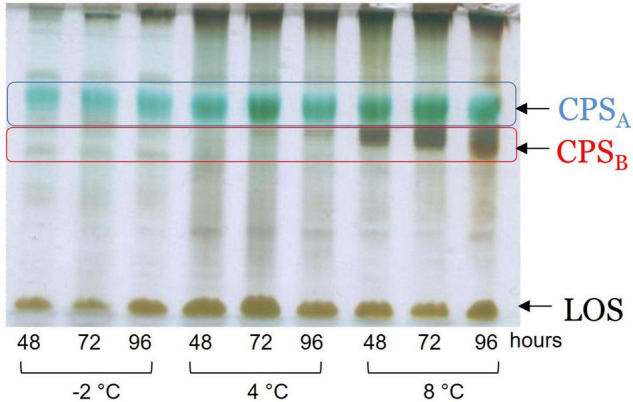
DOC-PAGE of the water extract from *Colwellia* 34H cell cultivation at −2, 4, and 8°C.

Since the monosaccharides constituting the CPS_*A*_, the CPS_*B*_, and the LOS are almost the same, it is difficult to correlate the production of the capsular polysaccharides (CPSs) in response to the temperature changes. Therefore, the extracts were analyzed by 14% DOC-PAGE and visualized after Alcian Blue and silver staining (Figure 3). Moving from −2 to 8°C, together with fast-migrating bands typical of the LOS, the electrophoretic profile showed bands at high molecular masses due to the presence of the CPS_*A*_ and CPS_*B*_. The DOC-PAGE analysis revealed to be more suitable to describe the production and the relative abundance of the polymers, demonstrating that the polysaccharide production exhibited changes in response to the growth temperature. The cultivation at the very low temperature suggested that the CPS_*A*_ endowed with IRI activity is the only produced, confirming the key role of this capsular in the survival of the bacterium. The cultivation of *Colwellia* 34H to 8°C resulted in the production of both CPS_*A*_ and CPS_*B*_.

**SCHEME 2:**
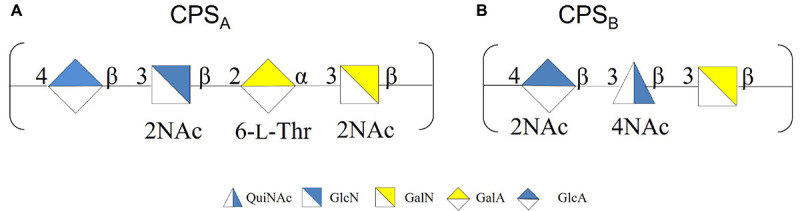
**(A)** CPS_*A*_ produced at −2, 4, and 8°C. **(B)** CPS_*B*_ produced at 8°C.

To confirm the primary structures of all the CPSs, all the extracts were purified from nucleic acid and LOS molecules through an enzymatic hydrolysis and a mild acid hydrolysis, respectively. The pure CPSs obtained after gel filtration chromatography were analyzed by ^1^H NMR (Supplementary Figure 4), confirming that the changing temperature did not affect either the production or the primary structure (Scheme 2).

### Medium Released Polysaccharides

**FIGURE 4:**
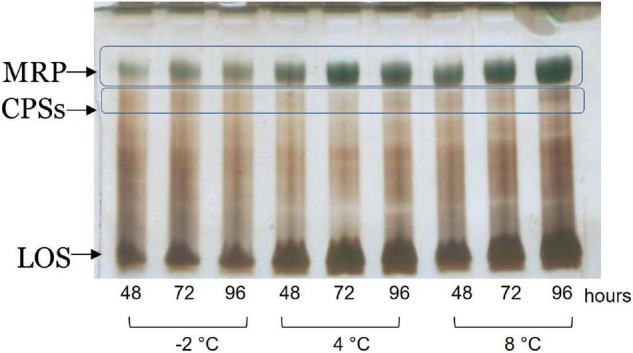
DOC-PAGE analysis of the dialyzed supernatant from *Colwellia* 34H.

The cell culture supernatants were dialyzed against water and freeze-dried. The monosaccharide composition revealed the presence of quinovosamine (QuiN) and galacturonic (GalA) residues, attributable to the MRP, as main components. Furthermore, the AMG analysis showed traces of GlcA, GalN, and GlcN, attributable to CPS_*A*_ and CPS_*B*_. The samples were analyzed by 14% DOC-PAGE, visualized after Alcian Blue and silver nitrate. The results of the DOC-PAGE experiment (Figure 4) suggested that moving from −2 to 8°C *Colwellia* 34H, the production of the EPS increased at higher temperatures.

**SCHEME 3:**
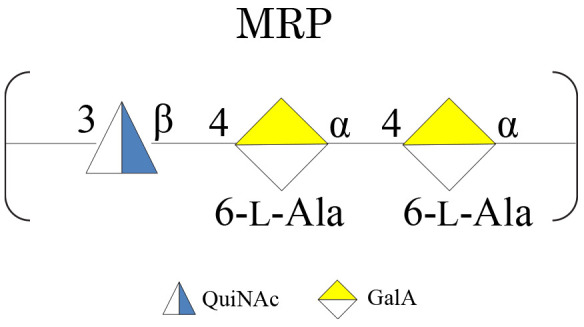
MRP isolated from *Colwellia* 34H at −2, 4, and 8°C.

The samples were subjected to mild acid hydrolysis and purified by S400 gel filtration chromatography column. The obtained polysaccharide fractions were analyzed by chemical analyses and NMR spectroscopy. The comparison of the ^1^H NMR spectra (Supplementary Figure 5) allowed confirming the structure of the MRP characterized from cultivation at 4°C in all the tested conditions (Scheme 3).

### Biofilm Production and Characterization

To investigate the capability of *Colwellia* 34H to form biofilm at different growth temperatures, the bacterium was grown in static conditions at −2, 4, and 8°C in Marine Broth medium, and biofilms were evaluated at different incubation times. The bacterium was able to form biofilm in all the tested conditions, and its formation occurs at the air—liquid interface forming pellicles or floating biofilms (Wimpenny et al., 2000). The amount of the biofilm formed in the diverse conditions was different; at −2°C, the amount of the biofilm produced by the bacterium was lower than that produced at 4 and 8°C. Biofilms produced by *Colwellia* 34H at different temperatures were further investigated by confocal laser scanning microscopy (CLSM) to analyze the structure and the biomass distribution.

**FIGURE 5:**
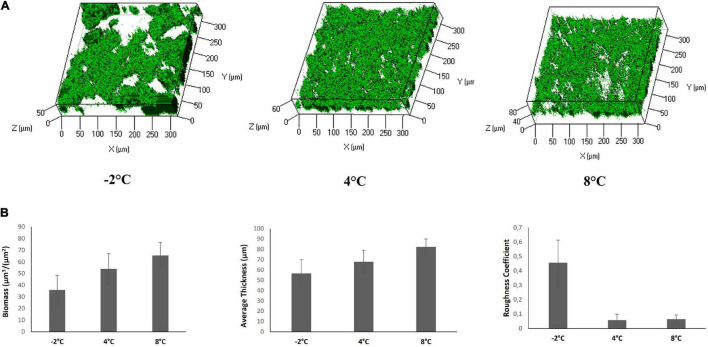
CLSM of *Colwellia* 34H biofilms in Marine Broth medium. **(A)** Z-stack (XYZ isosurface) analysis of *Colwellia* 34H biofilms at −2, 4, and 8°C. The bacteria were grown in chamber slides and then stained with LIVE/DEAD reagents. The green fluorescence (SYTO9) indicates viable cells. **(B)** COMSTAT quantitative analysis of the biomass, average thickness, and roughness coefficient of *Colwellia* 34H biofilms at −2, 4, and 8°C.

The biofilm structures were obtained using the live/dead staining, indicating viable cells by green fluorescence and red for dead (cell membrane damaged) bacteria (Figure 5A). The CLSM image stack data were further analyzed using the COMSTAT image analysis software package to evaluate the different parameters describing the biofilm structure (Figure 5B). The analysis revealed that the biofilm at −2°C proved to be less compact and structured worse than that produced at 4 and 8°C.

**FIGURE 6:**
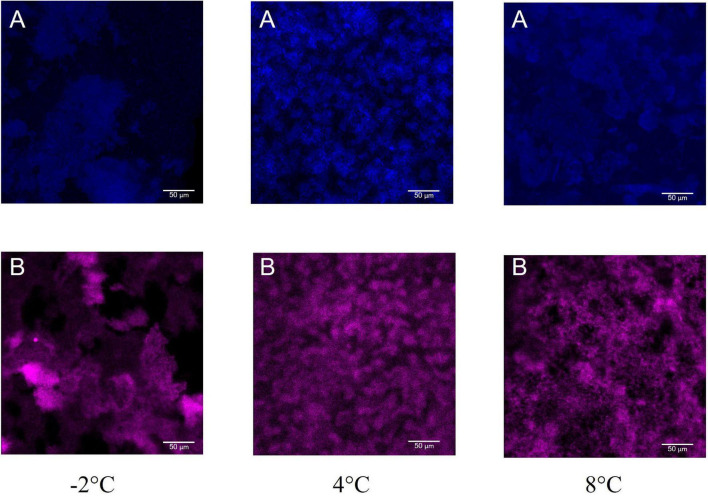
CLSM of *Colwellia* 34H biofilms at −2, 4, and 8°C. 2D images of *Colwellia* 34H biofilms. Biofilms were stained with wheat germ agglutinin (WGA), from Triticum vulgaris, FITC conjugated **(A)** that binds β-GlcNAc-(1→4)-β-GlcNAc-(1→4)-GlcNAc and Neu5Ac (sialic acid), and SYPRO^®^ Ruby Biofilm Matrix FilmTracer **(B)** used to reveal the proteins present in the biofilm matrix.

Moreover, the polysaccharide and the protein content of the biofilm matrix was explored by CLSM. A fluorescence lectin-binding analysis was carried out to obtain preliminary information on polysaccharide presence in *Colwellia* 34H biofilms. In particular, the biofilms obtained in the three studied conditions were stained with WGA, from Triticum vulgaris, FITC conjugated (Figure 6). WGA generally binds β-GlcNAc-(1→4)-β-GlcNAc-(1→4)-GlcNAc and Neu5Ac (sialic acid). SYPRO Ruby Biofilm Matrix FilmTracer was used to reveal the proteins present in the biofilm matrix, and the analysis revealed the presence of polysaccharides and proteins in all the tested conditions (Figure 6).

**FIGURE 7:**
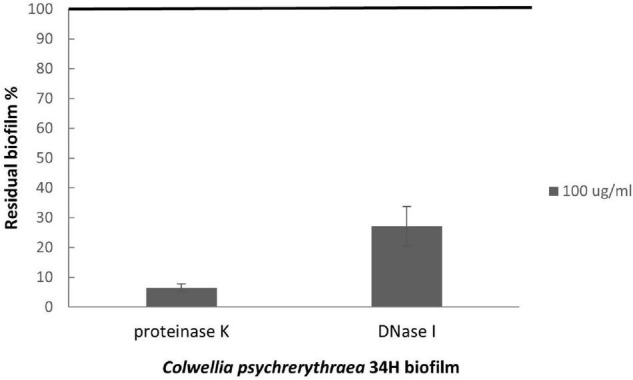
Analysis of the effect of proteinase K and DNase I on *Colwellia* 34H biofilm formation. *Colwellia* 34H biofilm formation obtained at 4°C for 96 h in the absence (100% biofilm obtained in absence of proteinase K or DNase I) and in the presence of proteinase K or DNase I at a concentration of 100 μg/ml. The data are reported as a percentage of the residual biofilm after the treatment. Each data point represents the mean ± the SD of six independent samples.

To better characterize the biofilm at 4°C, we analyzed the kinetics of the biofilm formation at 4°C (Supplementary Figure 6), and the highest biomass value was reached after 96 h. Therefore, the studies to assess the role of eDNA and proteins in the biofilm formation were performed, growing the cells in static conditions in the presence of either in the absence of DNase I or proteinase K for 96 h. The quantification of the biofilms obtained in these conditions (Figure 7) revealed that the treatment with DNaseI reduced the biofilm formation with respect to the biofilm obtained in the absence of the DNaseI. The proteinase K presence also affected the Arctic bacterium biofilm formation, suggesting the key role of proteins and eDNA during the biofilm formation of *Colwellia* 34H.

**FIGURE 8:**
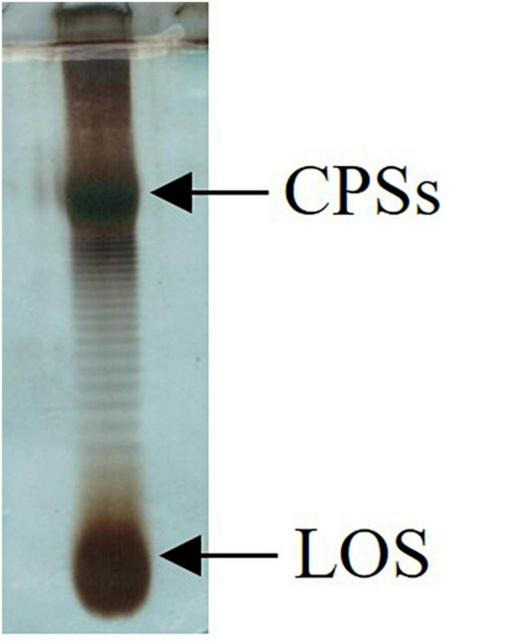
DOC-PAGE analysis of *Colwellia* 34H biofilm.

To investigate the biofilm in terms of polysaccharide composition, the air–liquid pellicles of *Colwellia* 34H grown in static condition at 4°C were recovered and analyzed by 14% DOC-PAGE experiment (Figure 8).

The gel visualized after Alcian Blue and silver nitrate staining showed the presence of one band at low molecular masses, corresponding to the LOS, and several bands at high molecular weight corresponding to the polysaccharide components. In agreement with the fact that in the biofilm the main biomass is attributable to the cells, glycosyl analysis performed on the *Colwellia* 34H biofilm confirmed the presence of the LOS and CPSs components. Moreover, the finding of ribose confirmed the occurrence of nucleic acids in the samples (data not shown).

## Discussion

*Colwellia* 34H is considered an excellent model for studying the lifestyle in cold environments (Methé et al., 2005) and is suitable for biotechnological applications since it produces cold-active enzymes with high catalytic efficiency at low temperatures (Park et al., 2017; Lee et al., 2019). Bacteria populating cold habitats evolved several strategies to avoid the freezing of their cells due to the low temperatures. The dropping of the temperature mainly affects the fluidity of the cellular membrane, thus compromising its functionality. The outer membrane of Gram-negative bacteria actively participates with all its constituents to reach this purpose. It is well known that the phospholipid composition changes vs. a higher amount of unsaturated fatty acids, an increased branching of acyl chains, and a decreased length. Consistent with these observations, the lipid A isolated from cold-adapted bacteria showed shortened acyl chains with respect to the mesophiles (Casillo et al., 2019a). This observation is mainly derived from the investigation of the temperature effect on mesophilic bacteria (Janoff et al., 1979; Kropinski et al., 1987), since a limited number of such studies are carried out on psychrophiles.

In the past, the effect of growth temperature on the LPS has been evaluated for the psychrotolerant *P. haloplanktis* TAC125. In that case, the change of the growth temperature among 4, 15, and 25°C resulted in two LOS structural variations: the presence of free lipid A at 4°C suggesting an incomplete biosynthesis in this condition, and a higher phosphate content from 4 to 25°C (Corsaro et al., 2004).

In the last few years, we focused our studies on the glycoconjugates produced by *Colwellia* 34H at 4°C, resulting in the isolation and characterization of very interesting molecules. In this paper, we analyzed the response of these glycoconjugates in terms of production and chemical structure by changing the growth temperature. The DOC-PAGE and chemical analyses of the extracted cells at −2, 4, and 8°C confirmed the rough nature of the LPS, with a slight difference in the yields. To compare the primary structure, the core oligosaccharides have been isolated and characterized. Our results demonstrated that no differences were found in the carbohydrate moiety of the lipooligosaccharides obtained from all the tested conditions.

The different amounts of some of the acyl chains obtained from the analyses of the fatty acid composition highlighted that the homeoviscous adaptation of the outer membrane is also attributable to the lipid A structures, as already reported for *Colwellia hornerae* and *Colwellia piezophila* (Sweet et al., 2015). At −2°C, increased production of monounsaturated fatty acids (C16:1) is observed, in agreement with the data available for other cold-adapted bacteria (Corsaro et al., 2004; Sweet et al., 2015).

The effect of temperature variation was also evaluated on the production of extracellular polysaccharides. The phenol/water extracts have been analyzed to compare the CPSs production. Moving from −2 to 8°C, the production and the structures of the CPS_*A*_ and MRP are confirmed, whereas the production of the CPS_*B*_ is verified to occur only at 8°C. Interestingly, this result indicated that *Colwellia* 34H was able to switch to a different polysaccharide biosynthesis instead of a mere downregulation of the polymers synthesized at low temperature.

The ability of bacteria to form biofilms in many environments is undoubtedly related to the selective advantage that the association offers; therefore, in this paper we also evaluated the ability of *Colwellia* 34H to form biofilm at different temperatures. The data demonstrated that this model organism responds to different temperatures producing a different amount of air–liquid biofilm. In all the tested conditions, the biofilm matrix obtained is quite similar, even if the analysis revealed that, at −2°C, the biofilm is less compact.

A more detailed investigation on the sugar content of biofilm at 4°C uncovered the absence of MRP molecules in the biofilm matrix, whereas further experiments revealed that the MRP molecules were present in the extracellular medium of the biofilm growth (data not shown). In many bacteria, the biofilm matrix contains MRPs (Karygianni et al., 2020), and in several strains, the MRPs have not only a structural role but also a functional one, for example, in *V. vulnificus* where MRPs are required for maturation of biofilm structures (Kim et al., 2009). The absence of MRP in the *Colwellia* 34H biofilm matrix could be related to the MRP role in cell cryoprotection; indeed, this function could be better accomplished if the MRP molecules are present in the culture medium where they can interact with the water molecules avoiding the formation of the tetrahydric geometry distinctive of ice crystals.

Since the analyses on *Colwellia* 34H biofilms revealed that the matrix contains not only sugar but also proteins and nucleic acids, we investigated the role of protein and eDNA on biofilm formation. Indeed, for some bacteria eDNA, besides its structural role, it is required for the initial step of biofilm formation, whereas in other bacteria, it plays a role during the transition from the attachment phase to the biofilm maturation phase (Okshevsky and Meyer, 2015). The reported data suggested that either eDNA or proteins seemed to be crucial for the biofilm formation of *Colwellia* 34H.

## Conclusion

In conclusion, we analyzed the cell culture of the model bacterium *C. psychrerythraea* 34H at three different temperatures to describe the variations in surface glycoconjugates and extracellular matrix glycopolymers. It is interesting to note that *C. psychrerythraea* 34H modulates in different ways all the analyzed glycoconjugates in response to different temperatures, by working on the fatty acid structural motifs or on the amount of the polymers. Finally, while the production of the CPS_*A*_ at all the temperatures is confirmed and its role in survival at low temperatures is clear, the physiological role of the CPS_*B*_ at 8°C is still under investigation.

## Data Availability Statement

The original contributions presented in the study are included in the article/Supplementary Material, further inquiries can be directed to the corresponding author/s.

## Author Contributions

AC performed the experiments, suggested critical parameters in design of experiments, and co-wrote the manuscript. CD’A performed the experiments and co-wrote the manuscript. EP and MT suggested critical parameters in the design of experiments and co-wrote the manuscript. MC designed the experiments, provided advice in the performance of experiments, and wrote the manuscript. All authors contributed to the article and approved the submitted version.

## Conflict of Interest

The authors declare that the research was conducted in the absence of any commercial or financial relationships that could be construed as a potential conflict of interest.

## Publisher’s Note

All claims expressed in this article are solely those of the authors and do not necessarily represent those of their affiliated organizations, or those of the publisher, the editors and the reviewers. Any product that may be evaluated in this article, or claim that may be made by its manufacturer, is not guaranteed or endorsed by the publisher.
