# Supplementary material for: Membrane and Extracellular Matrix Glycopolymers of Colwellia psychrerythraea 34H: Structural Changes at Different Growth Temperatures
Source: Front Microbiol. 2022 Feb 25;13:820714. doi: 10.3389/fmicb.2022.820714 (PMC8914368; doi:10.3389/fmicb.2022.820714)
Supplement: Supplementary file 1 [file Data_Sheet_1.docx]

Supplementary Material

Membrane and extracellular matrix glycopolymers of *Colwellia* *psychrerythraea* 34H: Structural changes at different growth temperatures

Angela Casillo^1*^, Caterina D’Angelo^1^, Ermenegilda Parrilli^1^, Maria Luisa Tutino^1^, Maria Michela Corsaro^1^

^1^ Department of Chemical Sciences, University of Naples “Federico II”, Complesso Universitario Monte S. Angelo, Via Cintia 4, 80126 Naples, Italy

**Table S1.** Cell and supernatant biomasses obtained from *Colwellia* 34H growth at -2, 4, and 8 °C.

|  | -2 °C | | | 4 °C | | | 8 °C | | |
| --- | --- | --- | --- | --- | --- | --- | --- | --- | --- |
|  | 48h | 72h | 96h | 48h | 72h | 96h | 48h | 72h | 96h |
| Cells (mg) | 83 | 109 | 112 | 107 | 131 | 141 | 103 | 107 | 95 |
| Supernatant (mg) | 228 | 210 | 247 | 162 | 154 | 156 | 238 | 178 | 174 |

**
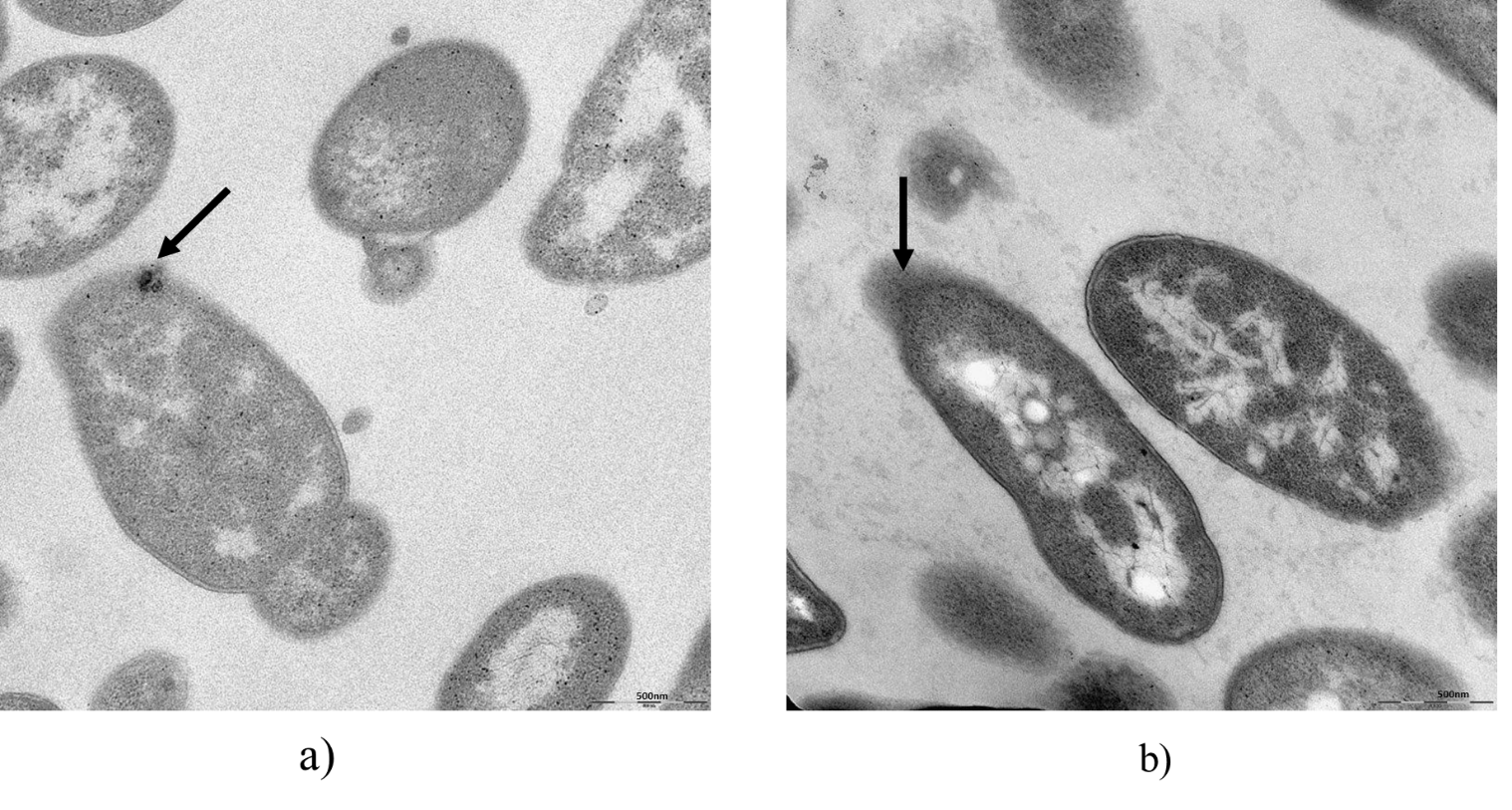
**

**Figure S1.** Transmission electron microscopy (TEM) images of thin sections of *Colwellia psychrerythraea* 34H grown at -2 °C (a) and 8 °C (b). The black arrows indicate the bacterial capsule.

**Figure S2.** MALDI of the LOS-OH from *Colwellia* 34H grown at -2 °C.

**Figure S3.** ^1^H NMR spectra of the OS from *Colwellia* 34H grown at -2 °C and 8 °C.

**Figure S4.** ^1^H NMR spectra of the CPS_A_ from *Colwellia* 34H grown at -2 °C and 8 °C.

**Figure S5.** ^1^H NMR spectra of the MRP from *Colwellia* 34H grown at -2 °C and 8 °C.

**Figure S6.** Analysis of *Colwellia* 34H biofilm formation at 4°C, the biofilms were analysed after 24h, 48h,72h,96h,120h with the crystal violet assay. Each data point was composed of six independent samples.
